# Methylglyoxal produces more changes in biochemical and biophysical properties of human IgG under high glucose compared to normal glucose level

**DOI:** 10.1371/journal.pone.0191014

**Published:** 2018-01-19

**Authors:** Mohd Adnan Khan, Zarina Arif, Mohd Asad Khan, Moinuddin, Khursheed Alam

**Affiliations:** 1 Department of Biochemistry, J.N. Medical College, Faculty of Medicine, Aligarh Muslim University, Aligarh, Uttar Pradesh, India; 2 Department of Biosciences, Jamia Millia Islamia, New Delhi, India; Rosalind Franklin University of Medicine and Science, UNITED STATES

## Abstract

Hyperglycaemia triggers increased production of methylglyoxal which can cause gross modification in proteins’ structure vis-a-vis function though advanced glycation end products (AGEs). The AGEs may initiate vascular and nonvascular pathologies. In this study, we have examined the biochemical and biophysical changes in human IgG under normal and high glucose after introducing methylglyoxal into the assay mixture. This non-enzymatic reaction mainly engaged lysine residues as indicated by TNBS results. The UV results showed hyperchromicity in modified-IgG samples while fluorescence data supported AGEs formation during the course of reaction. Shift in amide I and amide II band position indicated perturbations in secondary structure. Increase carbonyl content and decrease in sulfhydryl suggests that the modification is accompanied by oxidative stress. All modified-IgG samples showed more thermostability than native IgG; the highest Tm was shown by IgG-high glucose-MGO variant. Results of ANS, Congo red and Thioflavin T dyes clearly suggest increase in hydrophobic patches and aggregation, respectively. SEM and TEM images support aggregates generation in modified-IgG samples.

## 1. Introduction

During certain conditions, such as diabetes mellitus, the blood sugar level increases several fold than normal, which if not controlled may lead to kidney damage [1], neurological damage [2], cardiovascular damage [3,4], damage to the retina [5] and damage to the feet [6] and lungs [7]. The increased level of blood glucose starts forming covalent adducts with various proteins including IgG through a non-enzymatic process called glycation. Irrespective of target protein the glycation is ubiquitously accompanied by generation of free radicals.

During chronic hyperglycaemia increased production of methylglyoxal occurs which may result in excessive production of advanced glycation end products (AGEs) [8]. The AGEs are insoluble adducts that accumulate on the proteins with long half-life such as IgG, collagen, lens protein etc. [9] which impairs the protein normal function and finally ends up in pathological states. Three different types of AGEs have been reported; (i) Fluorescent cross linking AGEs such as pentosidine [10] and crossline [11], (ii) Non-fluorescent cross linking AGEs such as imidazolium dilysine cross links [12], (iii) Non-cross-linking AGEs such as pyrraline [13], N-carboxymethyllysine (CML) [14] etc.

It has been argued that glucose toxicity may be associated with increased formation of methylglyoxal [15] which is formed by the enzymatic and non-enzymatic elimination of phosphate from triose phosphates, like dihydroxyacetone phosphate which is a glycolytic intermediate [16]. Under normal physiology, the detoxification of methylglyoxal occurs by the glyoxylase system present in the cytosol of all mammalian cells [17]. The altered glucose metabolism in insulin–resistant states (such as obesity and type 2 diabetes mellitus) may lead to increased formation of methylglyoxal [18]. The degree of hyperglycaemia determines the concentration of methylglyoxal in plasma of diabetic patients.

The glyoxylase provide critical defence against methylglyoxal damage. However, for proper functioning of the enzyme reduced glutathione is required, which is depleted during hyperglycaemia due to excessive production of ROS which results in accumulation of methylglyoxal, the undisputed champion of glycation [16].

IgG is a glycoprotein of approximately 150 kDa mass and constitutes about 75% of the total plasma immunoglobulin [19]. Furthermore, excess glycation of IgG is likely to affect its role as a defender [20]. A spectroscopic study on IgG glycated by methylglyoxal and glyoxal has been reported with regard to structural disruptions [21]. Furthermore, IgG has quite long half-life of 25.8 days and possesses 80 lysine residues and thus it is a better target for glycating agents [22, 23]. Glycated IgG is highly immunogenic and auto-antibodies against glycated IgG have been reported in the sera of diabetic [24] and rheumatoid arthritis patients [25].

In this study, IgG has been modified with methylglyoxal under normal (5 mM) and high glucose (10 mM) concentrations for 7 days and characterized by an array of biophysical and biochemical techniques with a view to understand the effect of glucose crowding on the potential of methylglyoxal in causing glycation of IgG.

## 2. Materials and methods

### 2.1 Materials

Methylglyoxal (MGO), Protein-A-agarose pre-packed affinity column, Congo red (CR), Thioflavin T (ThT), 8-anilinonaphthalene-1-sulfonic acid (ANS), sodium dodecyl sulphate, dialysis tubing, 2,4,6-trinitrobenzene sulphonic acid (TNBS) and standard molecular weight marker were purchased from Sigma Chemical Company (St. Louis, MO, USA). D-glucose was purchased from Qualigens, India. Nitroblue tetrazolium (NBT) dye, 2,4-dinitrophenylhydrazine (DNPH) and silver nitrate were obtained from SRL, India. Acrylamide, bisacrylamide, ammonium persulphate and N,N,N’,N’-tetramethylethylene- diamine (TEMED) were purchased from Bio-Rad Laboratories, USA. All other reagents were of highest analytical grade available. The study protocol was approved by the Institutional Ethics Committee (IEC), Faculty of Medicine, Aligarh Muslim University, India. Furthermore, the blood sample was voluntarily donated by MAK and the same was used for IgG isolation. This was reported to the IEC.

### 2.2 Purification of human serum IgG on protein A-agarose affinity column

Decomplemented sera of healthy human subjects were placed on top of the Protein A-Agarose affinity column to purify IgG [26]. The purity of IgG was judged from absorbance ratio (278/251) of 2.0 or more. The homogeneity was checked on 7.5% non-reducing SDS-polyacrylamide gel and a single homogeneous band was found of pure IgG (Inset to Figure A in S1 File).

### 2.3 IgG modification by methylglyoxal under normal and high glucose

At the time of experiment, IgG (6.67 μM) was rapidly transferred in assay tubes containing 5 mM or 10 mM glucose and 6.67 μM MGO and incubated for 7 days at 37°C in capped vials. A small volume of assay mixtures was withdrawn every 12 h and subjected to NBT assay. At the end of incubation the contents were extensively dialyzed against PBS (pH 7.4) to remove unbound methylglyoxal/glucose. If needed, the dialysates were stored at -20°C.

### 2.4 UV-Vis spectroscopy

Absorption profiles of modified IgG were recorded on Shimadzu spectrophotometer (model UV-1700) in the wavelength range of 240–400 nm using quartz cuvette of 1 cm path length. Increase in absorbance at 280 nm (hyperchromicity) was calculated from the following equation:

$$\%\mathrm{Hyperchromicity}\;\mathrm{at}\;280\;\mathrm{nm}=\frac{\mathrm{Absorbance}\;\mathrm{of}\;\mathrm{modified}\;\mathrm{IgG}-\mathrm{Absorbance}\;\mathrm{of}\;\mathrm{native}\;\mathrm{IgG}}{\mathrm{Absorbance}\;\mathrm{of}\;\mathrm{modified}\;\mathrm{IgG}}X100$$ (1)

### 2.5 Fluorescence analysis

Changes in the fluorescence properties of IgG upon modification by MGO under normal and high glucose were studied on Shimadzu spectrofluorometer (RF-5301 PC). The samples were excited at 285 nm (specific for tryptophan) and the emission spectra were recorded in the wavelength range of 290–400 nm [27]. The decrease in the fluorescence intensity (F.I.) was calculated from the following equation:

$$\%\mathrm{decrease}\;\mathrm{in}\;F.I.=\frac{\mathrm{FI}\;\mathrm{of}\;\mathrm{native}\;\mathrm{IgG}-\mathrm{FI}\;\mathrm{of}\;\mathrm{modified}\;\mathrm{IgG}}{\mathrm{FI}\;\mathrm{of}\;\mathrm{native}\;\mathrm{IgG}}X100$$ (2)

AGE specific fluorescence was measured by exciting the samples at 370 nm. In this case the emission spectra were recorded in the wavelength range of 400–600 nm [28]. The increase in F.I. was calculated from the following equation:

$$\%\mathrm{Increase}\;\mathrm{in}\;F.I=\frac{\mathrm{FI}\;\mathrm{of}\;\mathrm{modified}\;\mathrm{IgG}-\mathrm{FI}\;\mathrm{of}\;\mathrm{native}\;\mathrm{IgG}}{\mathrm{FI}\;\mathrm{of}\;\mathrm{modifid}\;\mathrm{IgG}}X100$$ (3)

### 2.6 Quantitative estimation of ε-amino groups by TNBS

The estimation of free ε-amino groups was carried out in native and modified IgG samples with the help of 2, 4, 6-trinitrobenzenesulphonic acid (TNBS) reagent. TNBS specifically reacts under mild conditions with reactive ε-amino groups to form trinitrophenyl derivatives [29]. Briefly, 100 μl of 0.5% (w/v) TNBS was added to 0.5 ml of MGO-modified IgG samples and incubated for 1 h at 37°C. At the end of the incubation the samples were solubilised in 0.25 ml of 10% SDS followed by the addition of 0.1 ml of 1 N HCl. Absorbance was recorded at 420 nm and ε-amino groups were calculated using molar extinction coefficient of 19,200 cm^-1^mol^-1^. Furthermore, reduction in ε-amino groups of IgG due to methylglyoxylation was calculated from the following formula:

$$\begin{array}{l} Percent\;modification\;of\;IgG\;\varepsilon ‑amino\;group\\ =\frac{Amount\;of\;\varepsilon ‑amino\;group\;in\;native\;IgG-Amount\;of\varepsilon ‑amino\;group\;in\;modified\;IgG}{Amount\;of\;\varepsilon ‑amino\;group\;innative\;IgG}\times 100 \end{array}$$ (4)

### 2.7 Effective protein hydrophobicity

Binding of ANS dye to native and modified IgG was evaluated in terms of increase in fluorescence intensity. A fresh stock of the dye was prepared in distilled water and concentration was determined spectrophotometrically using molar extinction coefficient of 5000 M^-1^cm^-1^ at 350 nm [30]. The molar ratio of protein to ANS was adjusted to 1:50 and emission spectra were recorded in the wavelength range of 400–600 nm after excitation of the samples at 380 nm. Increase in ANS binding to modified IgG was calculated from the following equation:

$$\%\mathrm{Increase}\;\mathrm{in}\;F.I.=\frac{\mathrm{FI}\;\mathrm{of}\;\mathrm{modified}\;\mathrm{IgG}-\mathrm{FI}\;\mathrm{of}\;\mathrm{native}\;\mathrm{IgG}}{\mathrm{FI}\;\mathrm{of}\;\mathrm{modified}\;\mathrm{IgG}}X100$$ (5)

### 2.8 Determination of protein bound carbonyl

Carbonyl content of native and modified IgG samples was determined after reaction with DNPH [31]. The final absorbance was read at 360 nm and the carbonyl content was determined using molar extinction coefficient of 22,000 M^-1^cm^-1^. The result was expressed as nmol/mg protein.

### 2.9 FT-IR spectroscopy

FT-IR spectra were recorded on PerkinElmer FT-IR spectrometer (model; spectrum 2) in the wavenumber range of 1400 to 1800 cm^-1^ which covers the typical amide I and amide II regions [32]. On the platform of attenuated total reflection (ATR) device, 6.67 μM of native IgG and modified IgG sample (6.67 μM IgG modified with 6.67 μM MGO under 5 mM and 10 mM glucose) in 10 μl volume were placed and spectra were collected.

### 2.10 Determination of free sulfhydryls by Ellman’s reagent

The free sulfhydryl groups in native and modified IgG samples were determined by Ellman’s reagent [33]. Following solutions were prepared:

DTNB Stock: 50 mM sodium acetate in distilled water containing 2 mM DTNB

Tris Buffer: 1M Tris, pH 8.0

DTNB working reagent was prepared by mixing 100 μl of Tris buffer, 840 μl distilled water and 50 μl of DTNB stock. Ten μl of the sample was mixed with 990 μl of DTNB working reagent and incubated for 5 min at 37°C. Absorbance was recorded at 412 nm. The free sulfhydryl content was determined using molar extinction coefficient of 13,600 M^-1^cm^-1^.

### 2.11 Detection of Amadori adduct by NBT reagent

The MGO-modified IgG samples were subjected to NBT reduction assay for Amadori adduct (ketoamine) as described [34]. Samples (300 μl) were mixed with 3 ml of 100 mM sodium carbonate buffer (pH 10.35) containing 0.25 mM NBT and incubated at 37°C for 2 h and absorbance was read at 525 nm against distilled water. The Amadori adduct was calculated using molar extinction coefficient of 12,640 M^-1^cm^-1^ for monoformazan. Increase in fructosamine content (FC) was calculated from the following equation:

$$\%\mathrm{Increase}\;\mathrm{in}\;\mathrm{FC}=\frac{\mathrm{FC}\;\mathrm{of}\;\mathrm{modified}\;\mathrm{IgG}-\mathrm{FC}\;\mathrm{of}\;\mathrm{native}\;\mathrm{IgG}}{\mathrm{FC}\;\mathrm{of}\;\mathrm{modified}\;\mathrm{IgG}}X100$$ (6)

### 2.12 Detection of 5-hydroxymethylfurfural (HMF) in native and modified IgG samples

Formation of 5-hydroxymethylfurfural (HMF) from the Amadori product (ketoamine) of modified IgG was detected by thiobarbituric acid (TBA) reaction according to the method described by Ney *et al* [35]. Briefly, 1 ml each of native IgG and modified IgG samples were mixed with 1 ml of oxalic acid (1M) and incubated at 100°C for 2 h. Then, the protein in the assay mixture was removed by precipitation with 40% trichloroacetic acid. 0.25 ml of TBA (0.05 M) was added to 0.75 ml of protein free filtrate and incubated at 40°C for 40 min. The colour was read at 443 nm and amount of HMF was calculated using molar extinction coefficient of 40,000 cm^-1^mol^-1^.

### 2.13 Thermal denaturation studies

Effect of MGO modification on stability of IgG under normal and high glucose was ascertained from midpoint melting temperatures (Tm) of samples. All samples were subjected to heat induced melting from 20°C to 95°C at a rate of 1°C/min. The change in absorbance at 280 nm was recorded. Percent denaturation was calculated from the following equation:

$$\mathrm{Percent}\;\mathrm{denaturation}=\frac{A_{T}-A_{20}}{A_{\max}-A_{20}}X100$$ (7)

where,

A_T_ = Absorbance at temperature T°C
A_max_ = Final absorbance on the completion of denaturation (95°C)
A_20_ = Initial absorbance at 20°C

### 2.14 Detection of aggregates by Congo red (CR) and Thioflavin T (ThT)

Congo red is a non fluorescent dye. When it binds with amyloid fibrils/ protein aggregates it gives apple green birefringence due to expansion (delocalization) of the conjugated π-π electron system. The molar ratio of sample to Congo red was kept at 1:2 and the assay mixture was incubated for 30 min at room temperature prior to spectra recording in the wavelength range of 300–700 nm [36].

Native and modified IgG samples were incubated with ThT in a molar ratio of 1:2 for 60 min at 25°C [37]. The samples were excited at 435 nm and emission profiles were recorded. Increase in ThT binding was calculated from the following equation:

$$\%\mathrm{Increase}\;\mathrm{in}\;F.I.=\frac{\mathrm{FI}\;\mathrm{of}\;\mathrm{modified}\;\mathrm{IgG}-\mathrm{FI}\;\mathrm{of}\;\mathrm{native}\;\mathrm{IgG}}{\mathrm{FI}\;\mathrm{of}\;\mathrm{modified}\;\mathrm{IgG}}X100$$ (8)

### 2.15 Scanning electron microscopy

Fifty μl of sample was placed on a glass slide and then a cover slip was mounted. This was followed by overnight dehydration at 37°C and 50% humidity. The sample was gold platedand visualized underscanning electron microscope.The image was recorded with the help of JEOL JSM-6510LV microscope at an acceleration voltage of 15 kV and 1500 x magnification.

### 2.16 Transmission electron microscopy

The sample was fixed, dehydrated with ethanol and passed through propylene oxide. This was followed by grid preparation and coating with formvar film. Then electron of specific wavelength was passed through the grid and the transmitted image was processed with the help of iTEM and TIA software.

### 2.17 Statistical analysis

Data are presented as mean ± standard deviation. Statistical significance of the data was determined by Student’s–t test and a p-value of <0.05 was considered as significant.

## 3. Results

### 3.1 UV studies on IgG modified by methylglyoxal under normal and high glucose

**Fig 1:**
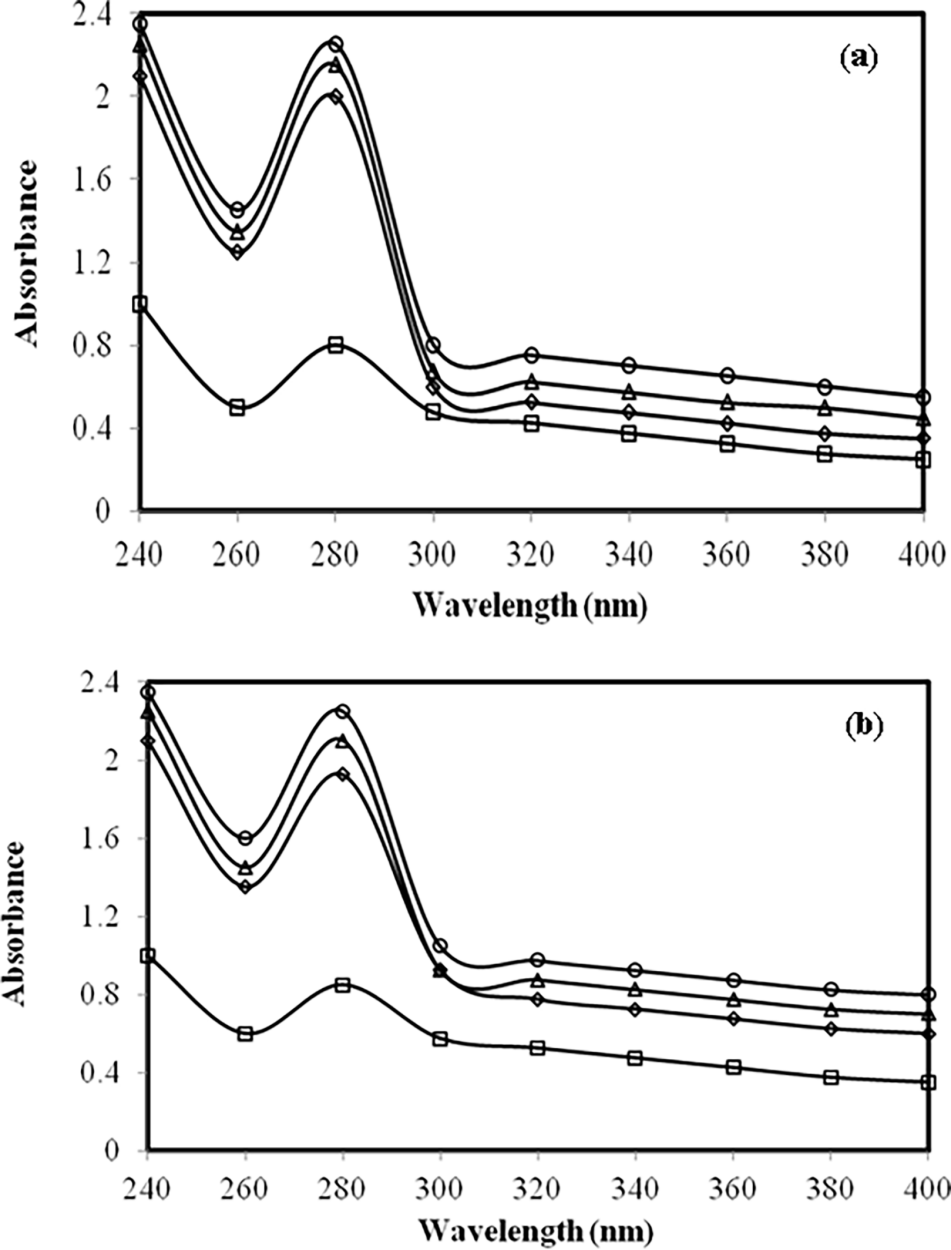
**(a) Absorbance study.** UV absorption spectra of native IgG (open square), IgG + 5 mM glucose (open rhombus), IgG + 5 mM glucose + 3.33 μM MGO (open triangle) and IgG + 5 mM glucose + 6.67 μM MGO (open circle) after 7 days.**(b) Absorbance study.** UV absorption spectra of native IgG (open square), IgG + 10 mM glucose(open rhombus), IgG + 10 mM glucose + 3.33 μM MGO (open triangle) and IgG + 10 mM glucose + 6.67 μM MGO (open circle) after 7 days.

Native IgG showed the λ_max_ at 280 nm (Figure A in S1 File). However, when IgG was incubated with normal glucose concentration (5 mM) for 7 days, we observed large hyperchromicity (Fig 1A). Furthermore, co-incubation with different concentrations of methylglyoxal caused further increase in hyperchromicity. A similar spectral pattern was observed with high glucose (10 mM) (Fig 1B). A daily record of change in absorbance of different samples has been shown in Table 1.

**Table 1:** Effect of time on 280 nm absorbance of native IgG and its modified counterparts.

Sample	Absorbance at 280 nm								
	(0 h)	(24 h)	(48 h)	(72 h)	(96 h)	(120 h)	(144 h)	(168 h)	(192 h)
Native IgG	0.80 (0.0)	0.82 (0.0)	0.84 (0.0)	0.85 (0.0)	0.86 (0.0)	0.87 (0.0)	0.88 (0.0)	0.88 (0.0)	0.88 (0.0)
IgG + 5 mMglucose	1.4 (42.85)	1.46 (43.83)	1.54 (45.45)	1.61 (47.20)	1.72 (50.00)	1.8 (51.66)	1.84 (52.17)	1.91 (53.92)	1.86 (52.68)
IgG + 10 mM glucose	1.5 (46.66)	1.55 (47.09)	1.62 (48.14)	1.7 (50.00)	1.8 (52.22)	1.9 (54.21)	1.96 (55.10)	2.01 (56.21)	1.9 (55.55)
IgG + 5 mM glucose +3.33 μM MGO	1.54 (48.05)	1.62 (49.38)	1.7 (50.58)	1.78 (52.24)	1.86 (53.76)	1.95 (55.38)	2.03 (56.65)	2.1 (58.09)	2.05 (57.07)
IgG + 5 mM glucose +6.67 μM MGO	1.64 (51.21)	1.74 (52.87)	1.81 (53.59)	1.91 (55.49)	1.99 (56.78)	2.06 (57.76)	2.13 (58.68)	2.2 (60.00)	2.16 (59.25)
IgG + 10 mM glucose +3.33 μM MGO	1.72 (53.48)	1.82 (54.94)	1.89 (55.55)	2.02 (57.92)	2.15 (60.00)	2.27 (61.67)	2.39 (63.17)	2.54 (65.35)	2.49 (64.65)
IgG + 10 mM glucose +6.67 μM MGO	1.82 (56.04)	1.94 (57.73)	2.1 (60.00)	2.17 (60.82)	2.29 (62.44)	2.49 (65.06)	2.64 (66.66)	2.75 (68.00)	2.67 (67.04)

Note: The data within parentheses indicate percent change in absorbance at 280 nm compared to native IgG.

### 3.2 Fluorescence studies

**Fig 2:**
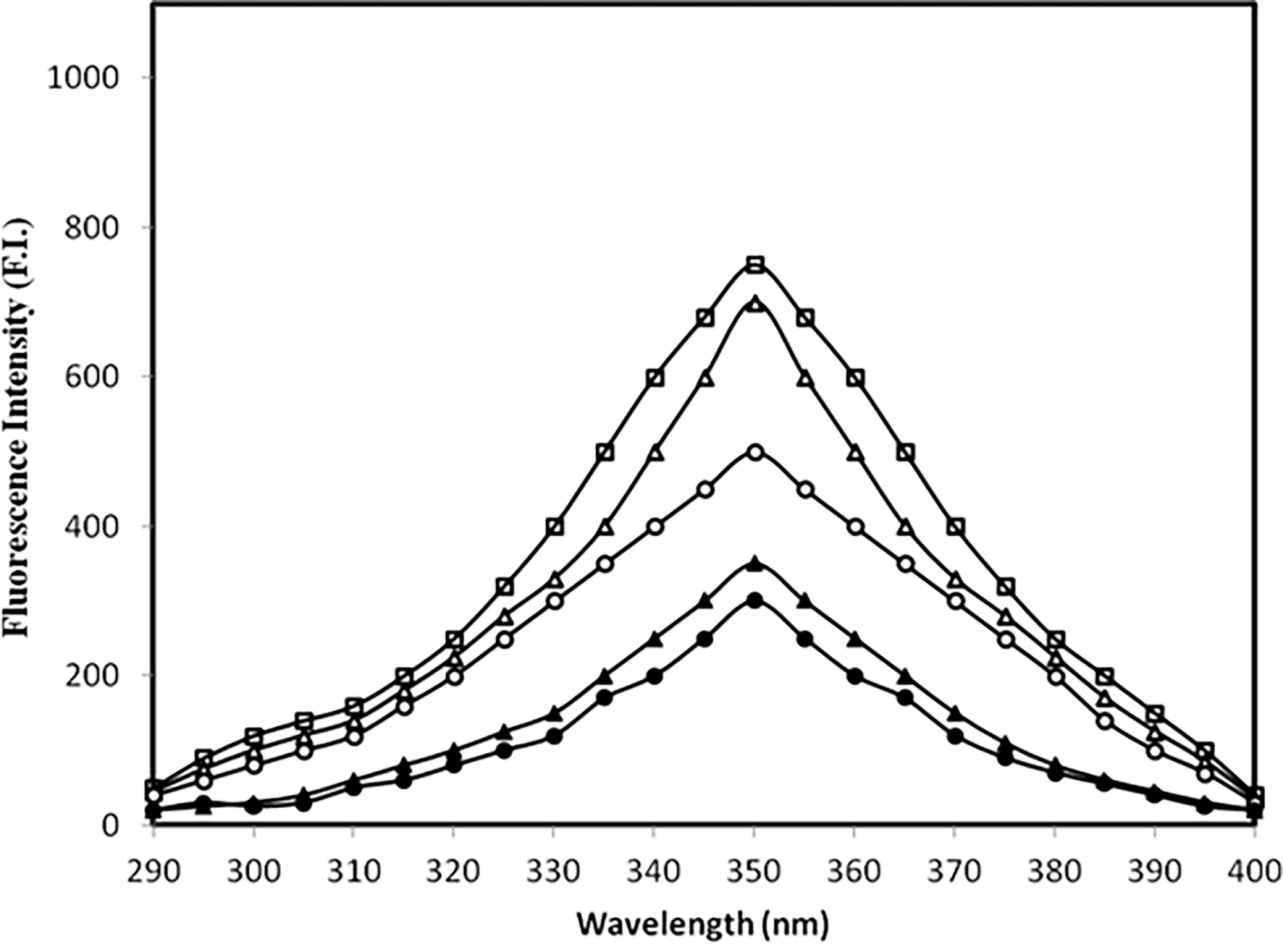
Fluorescence study. Emission profile of native IgG (open square), IgG + 5.0 mM glucose (open triangle), IgG + 5.0 mM glucose + 6.67 μM MGO (filled triangle), IgG + 10.0 mM glucose (open circle) and IgG + 10.0 mM glucose + 6.67 μM MGO (filled circle) after 7 days. All samples were excited at 285 nm for tryptophan fluorescence.

IgG’s tryptophan fluorescence is influenced by changes in its surrounding/microenvironment and that parameter was taken into consideration to analyse the impact of IgG modification by methylglyoxal under normal and high glucose [38]. All samples were excited at 285 nm and the results are shown in (Fig 2). The high glucose caused appreciable decrease in tryptophan fluorescence which further decreased in presence of MGO. The detailed results are summarized in Table 2.

**Table 2:** Effect of time on tryptophan specific fluorescence of various samples.

Sample	Fluorescence intensity (F.I.)							
	(0 h)	(24 h)	(48 h)	(72 h)	(96 h)	(120 h)	(144 h)	(168 h)
Native IgG	980	950	917	880	848	800	776	760
IgG + 5 mM glucose	934 (4.69)	900 (5.26)	866 (5.56)	824 (6.36)	792 (6.60)	745 (6.87)	721 (7.08)	700 (7.89)
IgG + 10 mM glucose	912 (7.45)	872 (8.21)	816 (11.01)	743 (15.56)	706 (16.74)	657 (17.87)	540 (30.41)	490 (35.52)
IgG + 5 mM glucose + 6.67 μM MGO	700 (28.57)	646 (32.00)	606 (33.91)	576 (34.54)	516 (39.15)	442(44.75)	396 (48.96)	320 (57.89)
IgG + 10 mM glucose + 6.67 μM MGO	600 (38.77)	564 (40.63)	514 (43.94)	478 (45.68)	422 (50.23)	376 (53.00)	300 (61.34)	260 (65.78)

Note: The data within parentheses indicate percent decrease in F.I. compared to native IgG.

**Fig 3:**
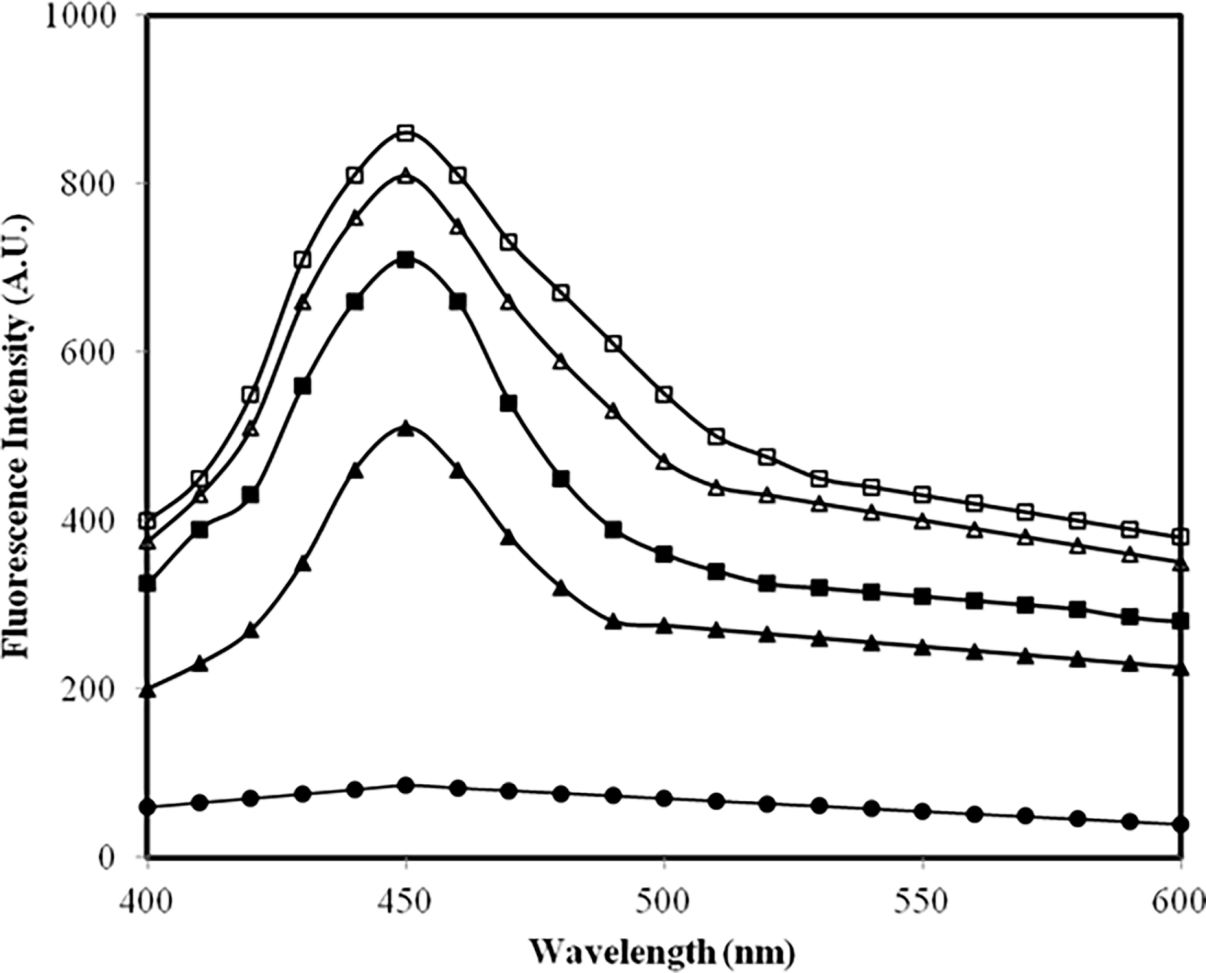
AGEs specific fluorescence assay. Emission profile of native IgG (filled circle), IgG + 5.0 mM glucose (filled triangle), IgG + 5.0 mM glucose + 6.67 μM MGO (open triangle), IgG + 10.0 mM glucose (filled square) and IgG + 10.0 mM glucose + 6.67 μM MGO (open square) after 7 days. All samples were excited at 370 nm for AGEs.

Likely formation of fluorogenic AGEs in samples was assessed by feeding 370 nm [39] wavelength and emission profiles were recorded (Fig 3). Native IgG was devoid of AGEs. Under high glucose concentration AGEs formation was substantially high which further enhanced in presence of methylglyoxal. The results are summarized in Table 3.

**Table 3:** Effect of time on AGEs specific fluorescence of various samples.

Sample	Fluorescence intensity (F.I.)							
	(0 h)	(24 h)	(48 h)	(72 h)	(96 h)	(120 h)	(144 h)	(168 h)
Native IgG	55	65	75	85	85	85	85	85
IgG+ 5 mM glucose	100 (45.00)	130 (50.00)	250 (70.00)	320(73.43)	380 (77.63)	440 (80.68)	480 (82.29)	510 (83.33)
IgG+ 10 mM glucose	125 (56.00)	200 (67.50)	340 (77.94)	420 (79.76)	480 (82.29)	575 (85.21)	640 (86.71)	700 (87.85)
IgG + 5 mM glucose + 6.67 μM MGO	250 (78.00)	350 (81.42)	440 (82.95)	530 (83.96)	615 (86.17)	680 (87.50)	740 (88.51)	800 (89.37)
IgG + 10 mM glucose + 6.67 μM MGO	325 (83.07)	450 (85.55)	595 (87.39)	690 (87.68)	720 (88.19)	760 (88.81)	810 (89.50)	860 (90.11)

**Note:** (i) The data within parentheses indicate percent increase in F.I. compared to native IgG. (ii) All samples were excited at AGEs specific wavelength of 370 nm.

### 3.3 Estimation of ε-amino groups in IgG samples modified by methylglyoxal under normal and high glucose

As shown in Figure B in S1 File. addition of 5 mM (low) glucose to IgG has consumed some ε-amino moieties and it came down from 230.01±1.72 nmol/mg in native IgG to 200.221±1.54 nmol/mg in IgG containing 5 mM glucose. Addition of methylglyoxal further decreased the ε-amino moieties to 111.78±0.82 nmol/mg [40]. Furthermore, at 10 mM (high) glucose, the consumption of ε-amino group increased and methylglyoxal addition further engaged the ε-amino group.

### 3.4 Effective protein hydrophobicity

**Fig 4:**
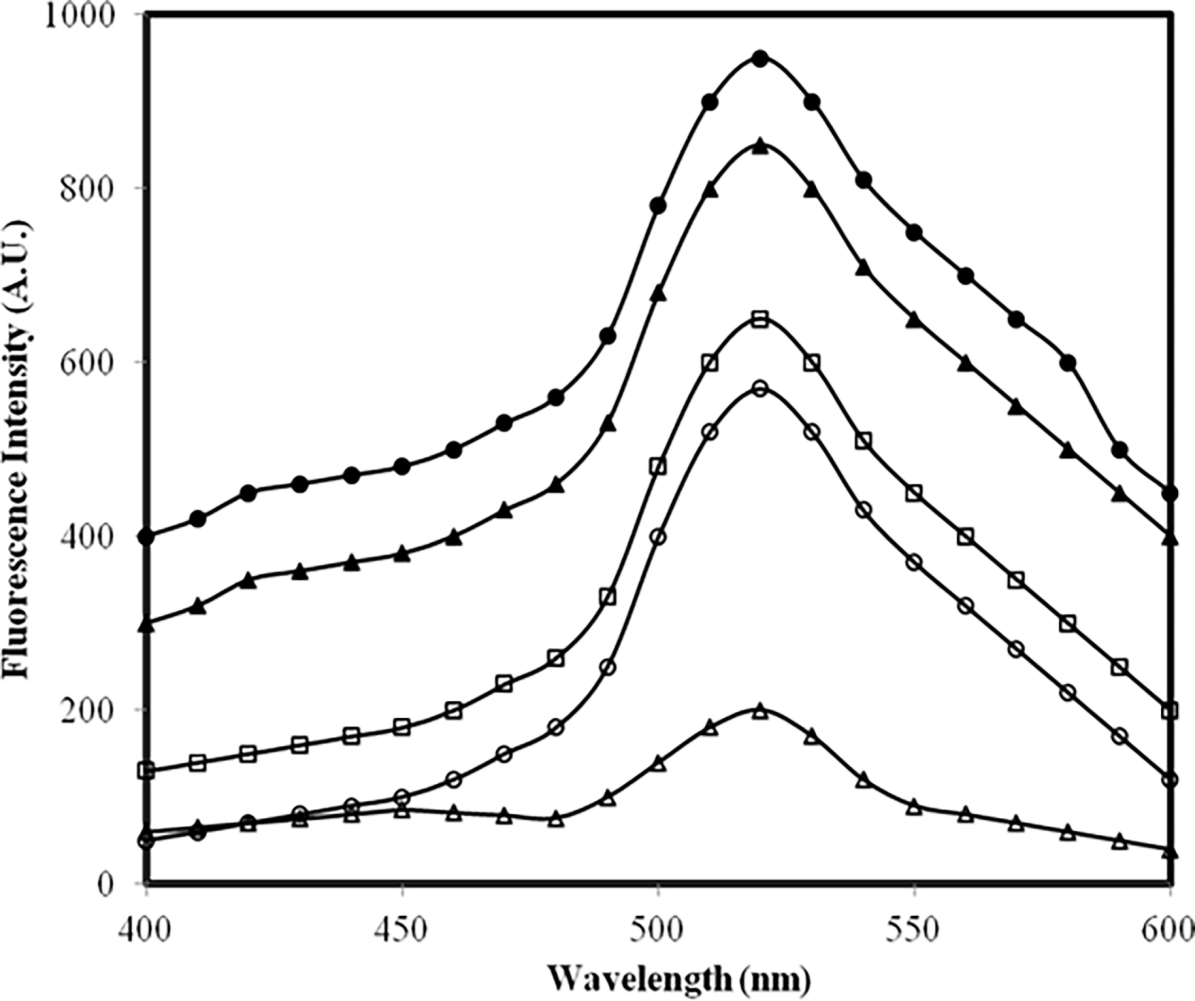
ANS fluorescence assay. Emission profile of ANS mixed with: native IgG (open triangle), IgG + 5.0 mM glucose (open circle), IgG + 10.0 mM glucose (open square), IgG + 5.0 mM glucose + 6.67 μM MGO (filled triangle) and IgG + 10.0 mM glucose + 6.67 μM MGO (filled circle). All samples were excited at 380 nm.

Profiles in Fig 4 suggest that methylglyoxal has led to exposure of more hydrophobic moieties/patches in IgG under high glucose compared to low glucose [41].

### 3.5 Estimation of protein carbonyl

**Fig 5:**
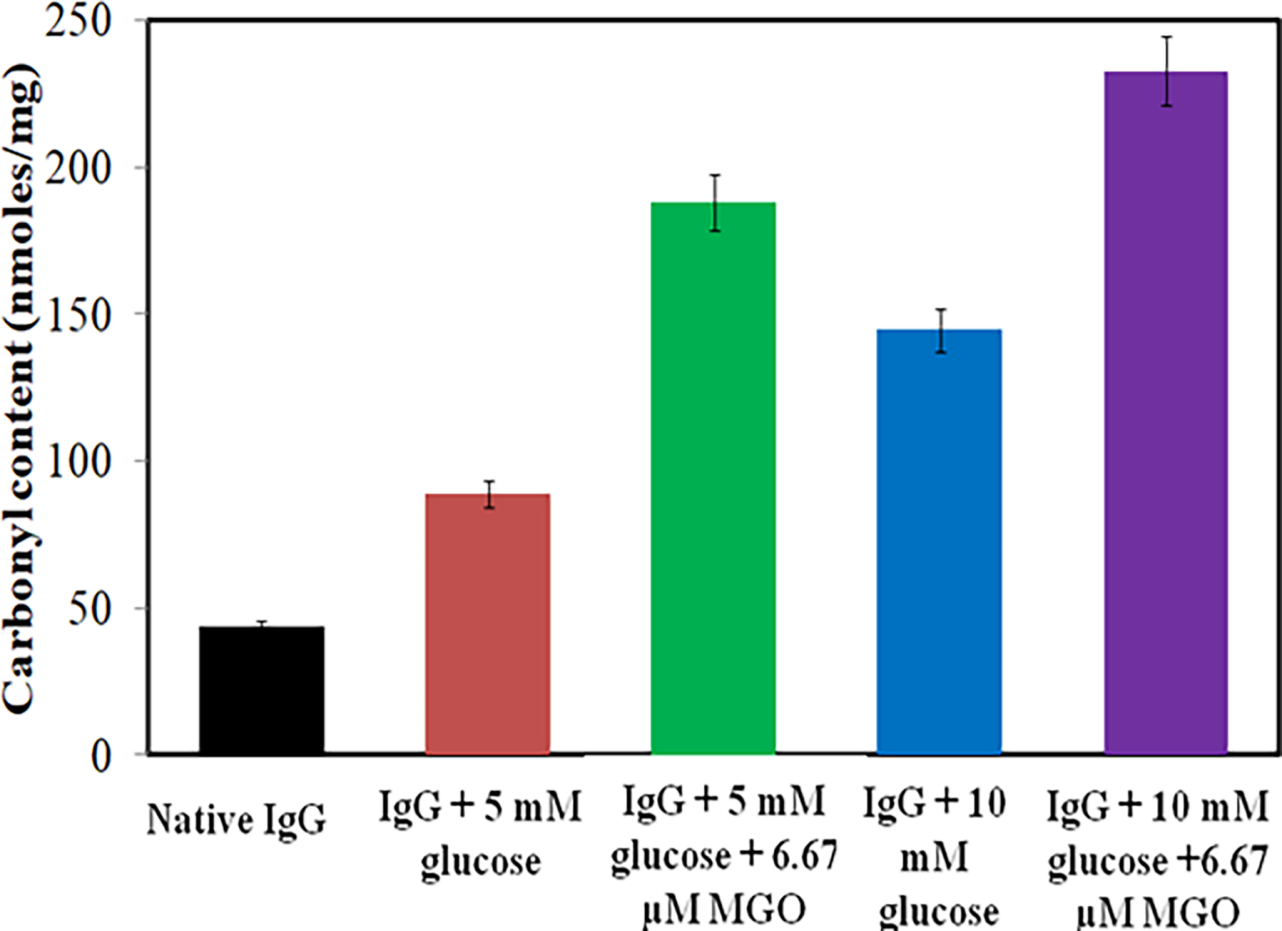
DNPH assay. Carbonyl content in native IgG (black), IgG + 5.0 mM glucose (red), IgG + 5.0 mM glucose + 6.67 μM MGO (green), IgG + 10.0 mM glucose (blue) and IgG + 10.0 mM glucose + 6.67 μM MGO (purple). Each bar represents the mean ± S.D. of three independent assays in similar experimental conditions. * p < 0.05 was considered as statistically significant as compared to native IgG with each modified group.

Carbonyls, biomarker of the oxidative stress [42], are outcome of the oxidation of lysine, arginine, threonine and proline residues. Upon reaction with dinitrophenylhydrazine, carbonyls form 2,4-dinitrophenylhydrazone and measured at 360 nm. The average carbonyl content of native IgG was found to be 43.56±0.97 nmol/mg of IgG (Fig 5). Both glucose and methylglyoxal produced stress on protein and lead to 2–5 fold increase in carbonyl.

### 3.6 FT-IR recording

**Fig 6:**
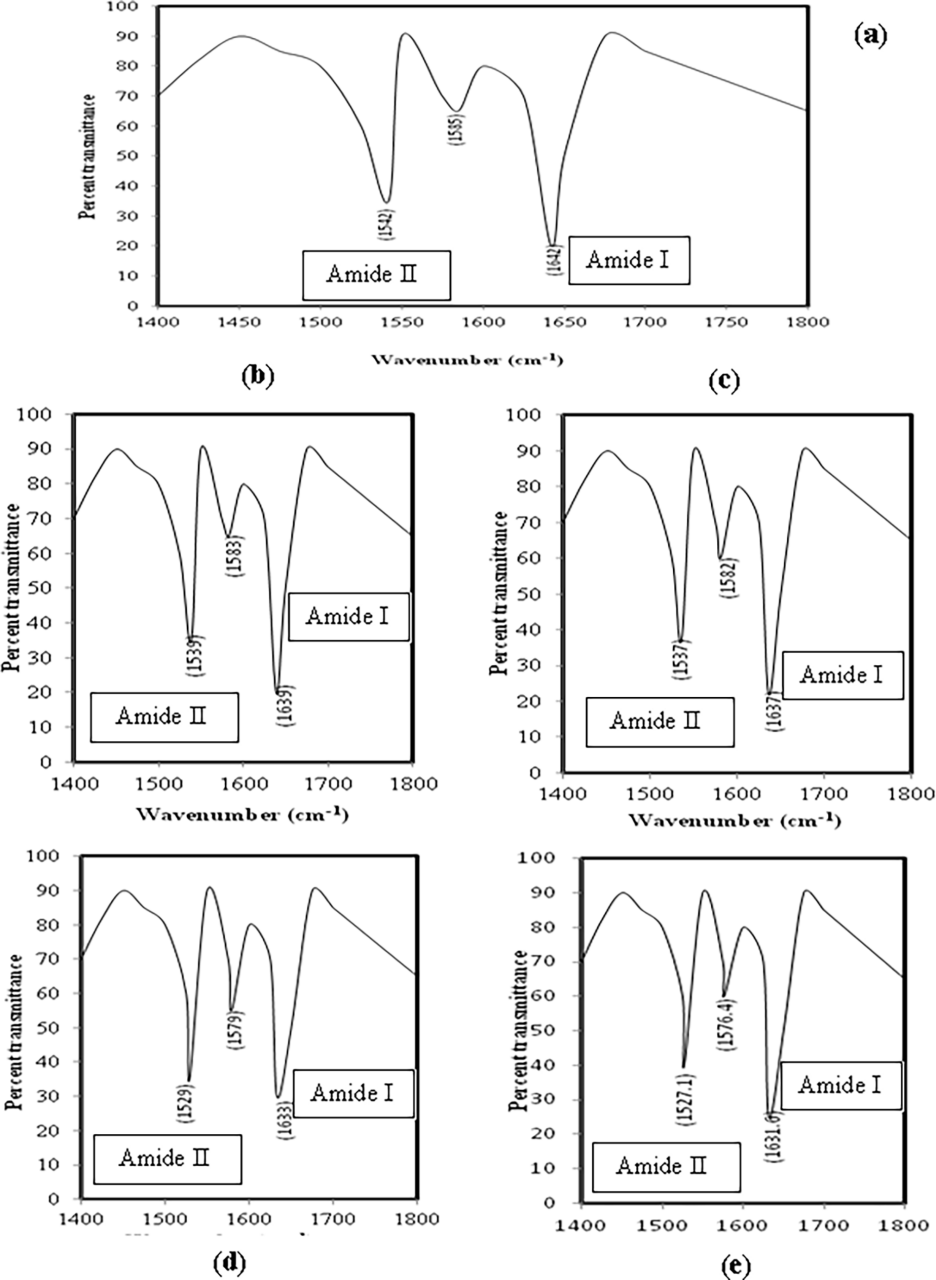
**(a) FT-IR study.** FTIRprofile of native IgG. **(b-e) FT-IR study.** FTIRprofiles of IgG + 5 mM glucose (b), IgG + 10 mM glucose (c), IgG + 5 mM glucose + 6.67 μM MGO (d) and IgG + 10 mM glucose + 6.67 μM MGO (e).

Changes in the secondary structure of proteins due to stretching and bending of bonds in peptide backbone can be examined by FT-IR spectroscopy. In case of native IgG the amide I band is seen between wave number 1624 and 1642 cm^-1^ while amide II band is located between 1510 and 1550 cm^-1^ [43]. The FT-IR spectra of native IgG, glucose modified IgG and MGO-glucose-modified IgG have been shown in (Fig 6A–6E). The changes in the vibrations of positions of Amide I and Amide II band in Fig 6B–6E is a clear indication of band stretching and/or bending during the modification. The data has been compiled in Table 4.

**Table 4:** FTIR band characteristics of native and modified IgG preparations.

Sample	Band type with wavenumber		
	Amide I	Amide II	Extra band
Native IgG	1642	1542	1585
IgG + 5 mM glucose	1639	1539	1583
IgG + 10 mM glucose	1637	1537	1582
IgG + 5 mM glucose + 6.67 μM MGO	1633	1529	1579
IgG + 10 mM glucose + 6.67 μM MGO	1631.6	1527.1	1576.4

### 3.7 Estimation of free sulfhydryl

Modifications induced by glucose and/or MGO on protein is generally accompanied by oxidative stress which leads to many biochemical changes. A change in the redox state of protein is also the consequence of oxidative stress, and sulfhydryl estimation by Ellman’s reagent is an authentic parameter of such an event. As shown in Figure C in S1 File the free sulfhydryl content in IgG decreased more under pathological concentration of glucose (10 mM) as compared to physiological level (5 mM). Furthermore, in presence of MGO the decrease in free sulfhydryls is elevated compared to what was observed with glucose alone. It simply suggests that the redox imbalance created in IgG by glucose got augmented in presence of methylglyoxal.

### 3.8 Nitrobluetetrazolium reduction assay for determination of early glycation products (Amadori products) in native and MGO-modified IgG in presence of glucose

**Fig 7:**
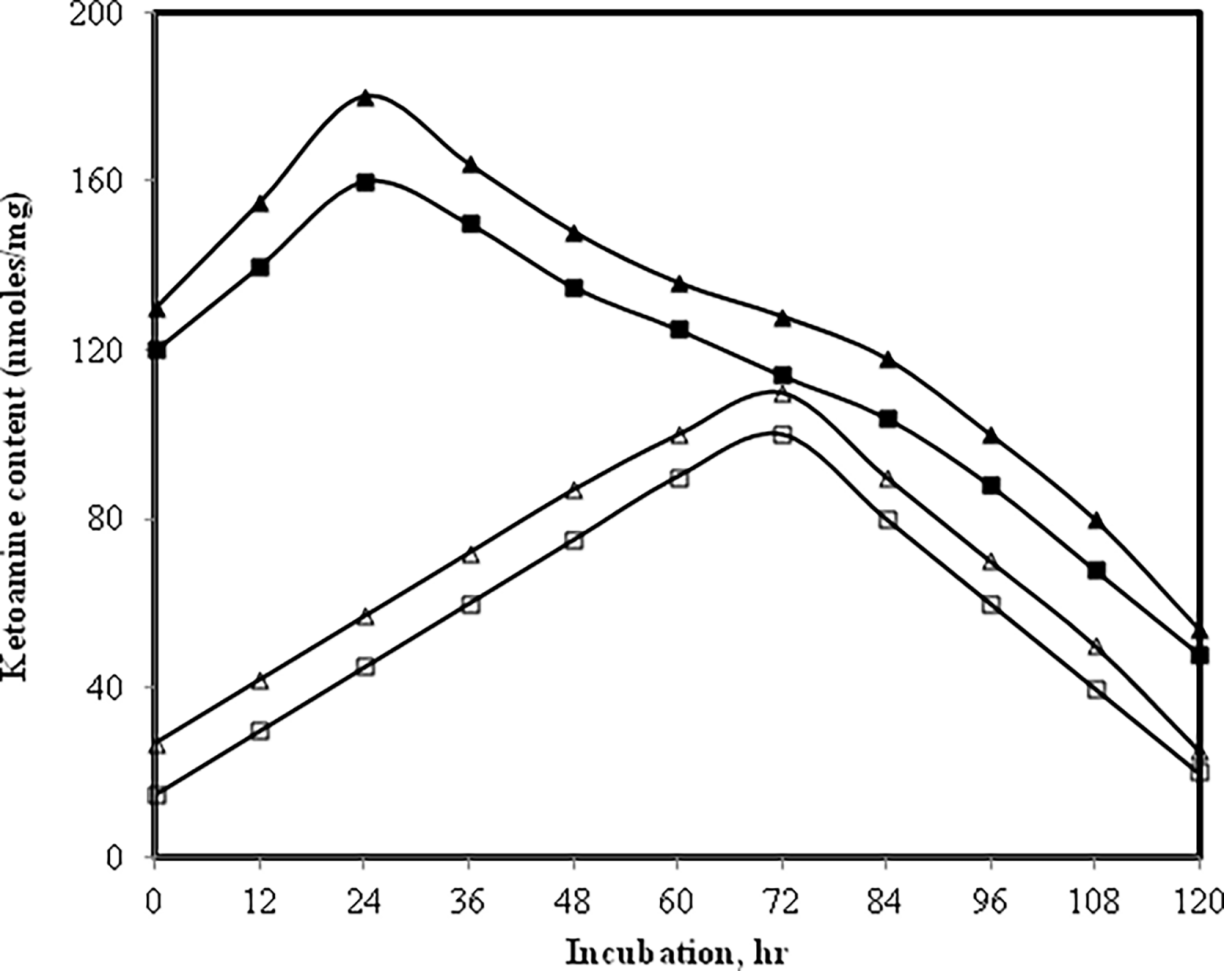
NBT assay. Level of Amadori adducts in IgG + 5 mM glucose (open square), IgG + 10 mM glucose (open triangle), IgG + 5 mM glucose + 6.67 μM MGO (filled square) and IgG + 10 mM glucose + 6.67 μM MGO (filled triangle).

NBT reduction assay was carried out on IgG modified by methylglyoxal under normal and high glucose to work out the optimum time required for optimum formation of Amadori products [44]. It was observed that in IgG samples containing normal or high glucose but devoid of methylglyoxal the Amadori formation completes by 72 h (Fig 7). However, in presence of methylglyoxal the time required for the formation of optimum amount of Amadori products was just 24 h. Furthermore, we also observed that there was high yield of Amadori products when methylglyoxal was present. With increase in incubation time the Amadori products slowly rearranged into AGEs.

### 3.9 Estimation of hydroxymethyl furfural (HMF) in native and modified-IgG samples

Treatment of Amadori adducts with weak acids (oxalic acid or acetic acid) yields HMF which react with thiobarbituric acid (TBA) and forms a derivative which shows the λ_max_ at 443 nm [45]. The assay measures the amount of ketoamine which is released upon hydrolysis as 5-hydroxymethyl furfural (HMF). The highest amount of HMF was released from IgG modified by methylglyoxal in high glucose (Figure D in S1 File).

### 3.10 Thermal denaturation profile of native and MGO-modified IgG in presence of glucose

**Fig 8:**
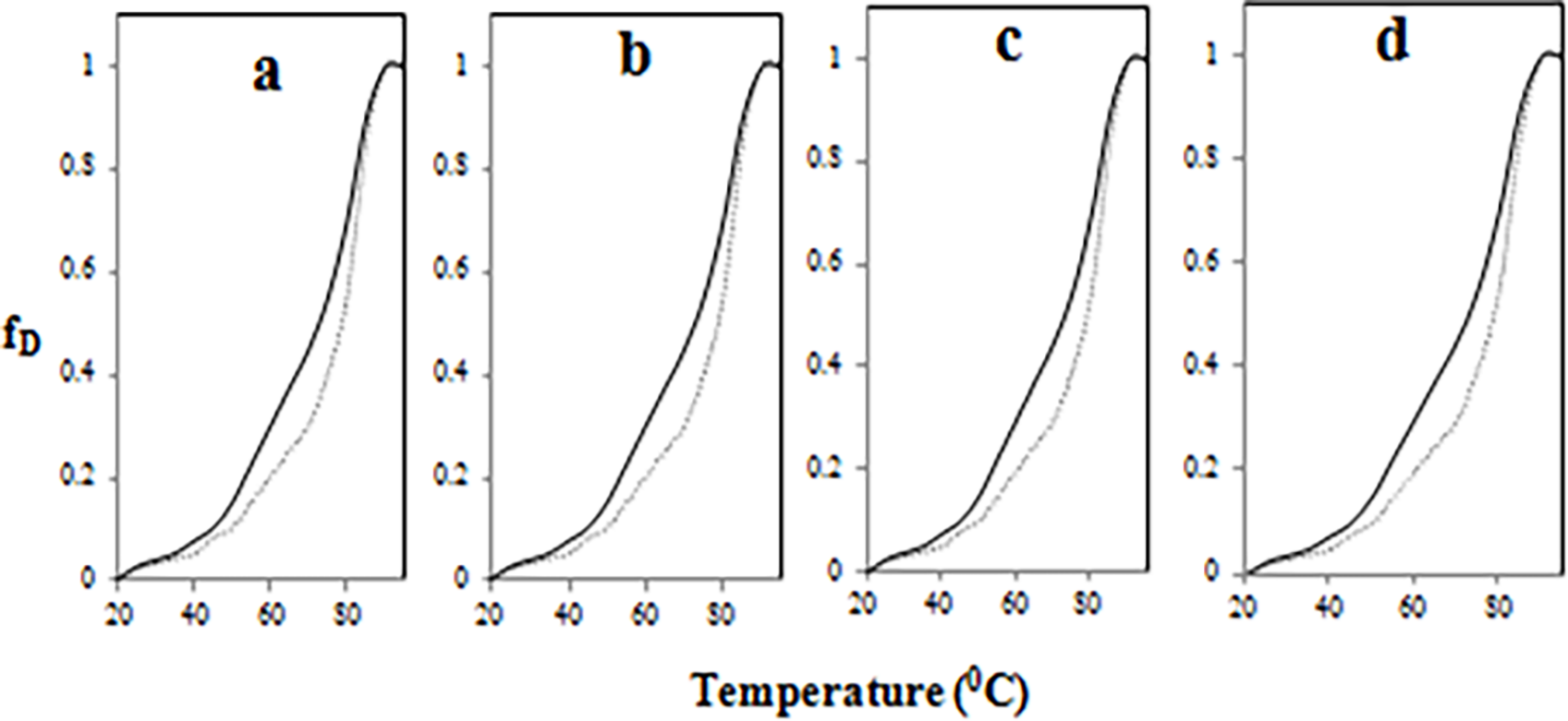
Thermal denaturation. Melting profiles of native (bold line in each), IgG + 5 mM glucose (dotted line in Fig a), IgG + 10 mM glucose (dotted line in Fig b), IgG + 5 mM glucose + 6.67 μM MGO (dotted line in Fig c) and IgG + 10 mM glucose + 6.67 μM MGO (dotted line in Fig d).

Heat-induced structural transitions in native and modified-IgG samples was monitored at 280 nm by heating at 1°C/min using Peltier device. The increase in absorbance at 280 nm was taken as a measure of denaturation. The melting temperature (Tm) of the native IgG was calculated to be 72.5°C (Fig 8A–8D). The data in (Fig 8A–8D) suggests that the modification introduced by glucose and/or methylglyoxal has lead to structural reorganization and the molecule (IgG) has gained stability.

### 3.11 Aggregates detection by Congo red and Thioflavin T

The λ_max_ of Congo red dye is significantly enhanced when bound by protein aggregates [46]. When native IgG was incubated with dye no meaningful change in λ_max_ was observed (Figure E in S1 File).However, under identical conditions the dye showed enhancement and shift in its λ_max_ when bound by glucose and MGO-modified counterparts of IgG. The findings indicate presence of aggregates. Further evidence of aggregates formation came from enhancement in the emission intensities of modified-counterparts of IgG mixed with Thioflavin T (ThT) (Figure F in S1 File). Thioflavin T has been used for aggregates detection [47].

### 3.12 SEM and TEM analysis of native and modified-IgG sample

**Fig 9:**
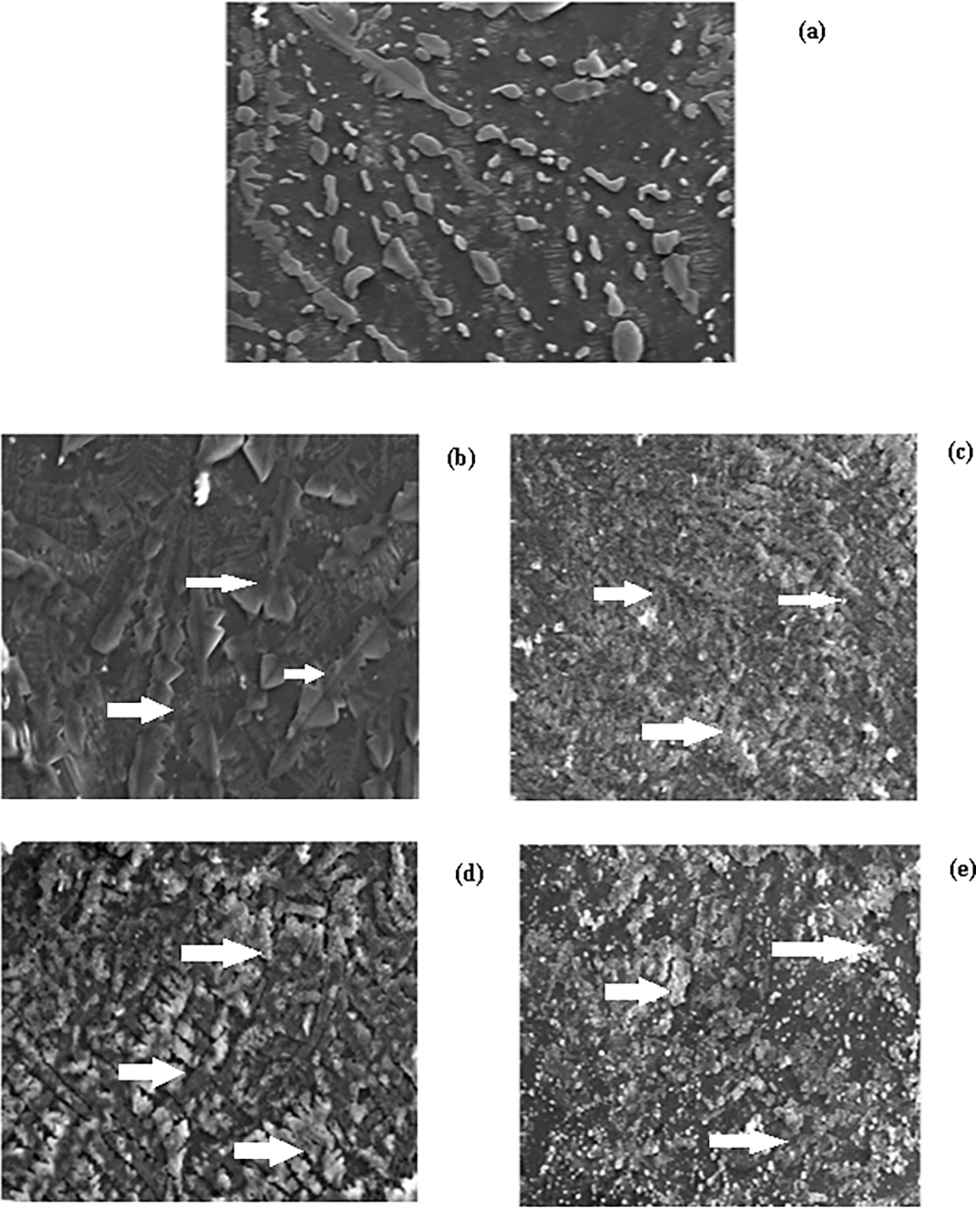
Scanning electron microscopy. SEM images of native IgG (a),IgG + 5 mM glucose (b), IgG + 5 mM glucose + 6.67 μM MGO (c),IgG + 10 mM glucose (d) and IgG + 10 mM glucose + 6.67 μM MGO (e). All magnification at 1500 x and at an acceleration voltage of 15 kV.

Native IgG visualized under scanning electron microscope (SEM) looked like a rod (Fig 9A), while IgG dissolved in normal and high glucose appeared as aggregates as shown by their morphology in SEM (Fig 9B–9D) and when treated with methylglyoxal the formation of aggregates got augmented and thus it appeared as large aggregates as revealed by SEM images (Fig 9C–9E). The findings suggest that methylglyoxylation of sweet IgG may led to aggregate formation, which was also shown by Congo red and Thioflavin T binding assay, respectively.

**Fig 10:**
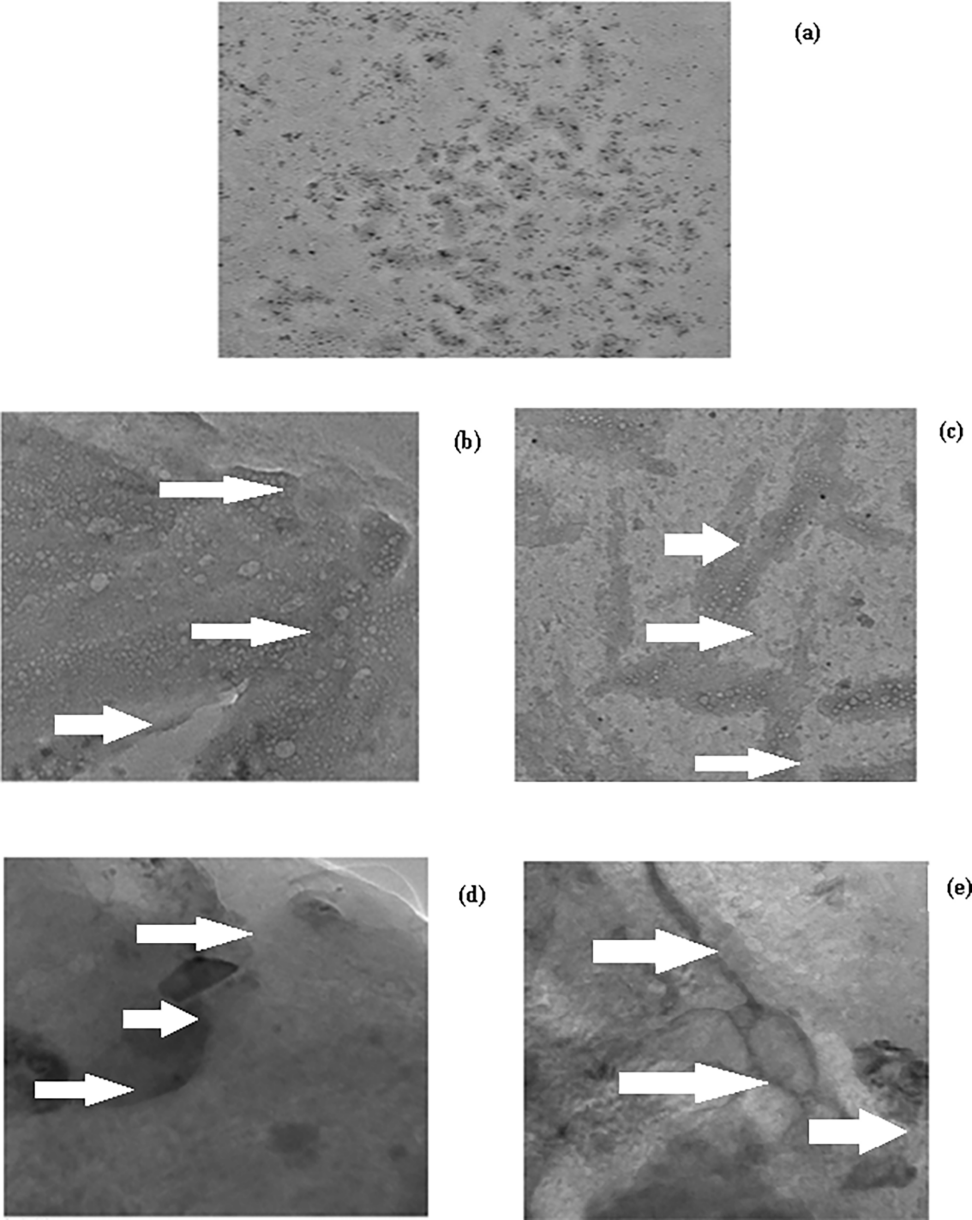
Transmission electron microscopy. TEM images of native IgG (a), IgG + 5 mM glucose (b), IgG + 5 mM glucose + 6.67 μM MGO (c), IgG + 10 mM glucose (d) and IgG + 10 mM glucose + 6.67 μM MGO (e). All magnification at 60000 x and at an acceleration voltage of 200 kV.

Furthermore, under transmission electron microscope the native IgG appeared as stretch of globules (Fig 10A), while low and high glucose versions of modified IgG appeared as extended branched protein networks with large surface area and amorphous and irregular in shape (Fig 10B–10D); branching and surface area of these aggregates further got extended in presence of methylglyoxal which shows increased formation of aggregates as also revealed by SEM images (Fig 10C–10E).

## 4. Discussion

Dicarbonyls, such as methylglyoxal and glyoxal, are excessively formed in diabetes mellitus patients. Upon reaction with proteins the dicarbonyls generate advanced glycation end product (AGEs) which may cause diabetic retinopathy, nephropathy, neuropathy, rheumatoid arthritis etc [48–51]. Participation of modified-IgG in autoimmune disorders (especially rheumatoid arthritis) has been reported by several authors [52].

IgG incubated with glucose and/or MGO showed decrease in ε-amino groups. This may be attributed to blocking of ε-amino groups by MGO. The hyperchromicities shown by modified-IgG samples in presence of normal glucose, high glucose and/or MGO may be due to enhanced exposure of chromophoric aromatic amino acid residues which were otherwise buried in case of native IgG [53]. Furthermore, the increased absorbance that we observed between 300–400 nm in modified IgG samples indicates aggregate formation [23]. Positive results with Congo red and ThT dyes and typical features observed under SEM and TEM strongly support aggregate formation. It has been suggested that the aggregates may lead to various pathological conditions, collectively known as proteopathies [54].

The observed quenching in tryptophan fluorescence of modified-IgG samples after its excitation at 285 nm may be due to modification of its microenvironment/ loss of tryptophan. A similar observation has been reported by Iram *et al* [55] when haemoglobin was modified by glyoxal. Furthermore, increase in emission intensity of modified-IgG samples after excitation at 370 nm suggest presence of fluorogenic AGEs, such as pentosidine, crossline etc. [56].

IgG modified by glucose and/or MGO generated Amadori products and was confirmed by NBT dye and HMF [57, 58]. This further underwent rearrangement reaction such as cyclization, condensation etc. and formed AGEs. Furthermore, increase in protein carbonyl and decrease in sulfhydryl suggests that the reaction has produced oxidative stress in the system [59, 60].

Changes in 2^0^ structure of modified-IgG samples was indicated by shift in position of amide I and amide II band position observed in FT-IR spectra which was due to C = O stretching and N-H bending bond vibration [61]. The thermostability shown by modified-IgG samples may be due to increase in disulphide/formation of cross links as a consequence of oxidative stress which occurred during the modification and reflected as increase in protein carbonyl and decrease in free sulfhydryl.

## 5. Conclusion

Results of the present study suggest that methylglyoxal can cause more damage to IgG in high glucose compared to low glucose and the damage can lead to aggregation. Since aggregates are immunogenic, some patients of diabetes mellitus may develop features of rheumatoid arthritis.

## Supporting information

S1 File **Fig A. Absorbance study.** UV absorption spectra of native IgG (closed square) isolated from a healthy human serum on protein A-agarose affinity matrix. **Inset:** SDS-gel photograph of purified IgG on 7.5% polyacrylamide gel. **Fig B. TNBS assay.** Estimation of ε-amino groups in native IgG (black bar), IgG + 5 mM glucose (red), IgG + 5 mM glucose + 6.67 μM MGO (green), IgG + 10 mM glucose (blue), and IgG + 10 mM glucose + 6.67 μM MGO (purple). Each bar represents the mean ± S.D. of three independent assays in similar experimental conditions. * p < 0.05 was considered as statistically significant as compared to native IgG with each modified group. **Fig C. DTNB assay.** Free sulfhydryl in native IgG (black bar), IgG + 5 mM glucose (red), IgG + 5 mM glucose + 6.67 μM MGO (green), IgG + 10 mM glucose (blue) and IgG + 10 mM glucose + 6.67 μM MGO (purple). Each bar represents the mean ± S.D. of three independent assays in similar experimental conditions. * p < 0.05 was considered as statistically significant as compared to native IgG with each modified group. **Fig D. HMF assay.** Hydroxymethylfurfural content in native IgG (black), IgG + 5 mM glucose (red), IgG + 5 mM glucose + 6.67 μM MGO (green), IgG + 10 mM glucose (blue) and IgG + 10 mM glucose + 6.67 μM MGO (purple). Each bar represents the mean ± S.D. of three independent assays in similar experimental conditions. * p < 0.05 was considered as statistically significant as compared to native IgG with each modified group. **Fig E. Congo Red binding assay.** Absorption profile of Congo red (open square) bound to: native IgG (open triangle), IgG + 5 mM glucose (open circle), IgG + 10 mM glucose (filled square), IgG + 5 mM glucose + 6.67 μM MGO (filled triangle) and IgG + 10 mM glucose + 6.67 μM MGO (filled circle).**Fig F. Thioflavin T binding assay.** Emission profile of Thioflavin T bound to native IgG (filled circle), IgG + 5 mM glucose (filled triangle), IgG + 10 mM glucose (filled square), IgG + 5 mM glucose + 6.67 μM MGO (open triangle) and IgG + 10 mM glucose + 6.67 μM MGO (open square). All samples were excited at 435 nm.
(DOCX)

## Abbreviations

IgG: Immunoglobulin G
MGO: Methylglyoxal
AGEs: Advanced glycation end-products
SEM: Scanning electron microscope
TEM: Transmission electron microscope
ANS: 8-anilinonaphthalene-1-sulfonic acid
DNPH: 2,4-Dinitrophenylhydrazine
FT-IR: Fourier transform infrared spectroscopy
DTNB: 5,5’-Dithiobis-(2-nitrobenzoic acid)
NBT: Nitroblue tetrazolium
