# Supplementary figures and images for: Methylglyoxal produces more changes in biochemical and biophysical properties of human IgG under high glucose compared to normal glucose level
Source: PLoS One. 2018 Jan 19;13(1):e0191014. doi: 10.1371/journal.pone.0191014 (PMC5774746; doi:10.1371/journal.pone.0191014)

**SUPPORTING INFORMATION FILE (S1 FILE)**

**
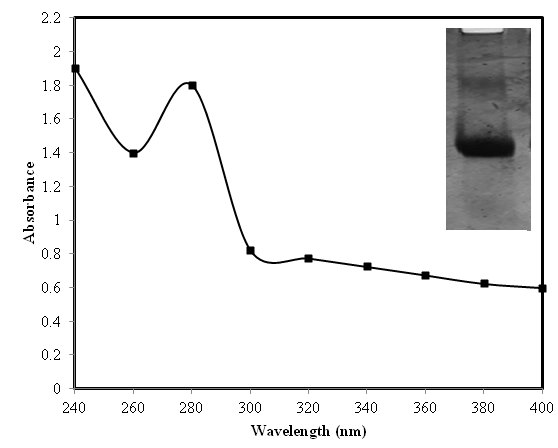
**

**Fig. A**

**
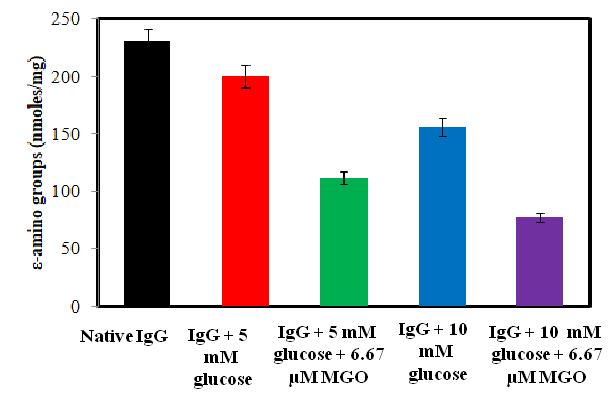
**

*

*

*

*

**Fig. B**

**
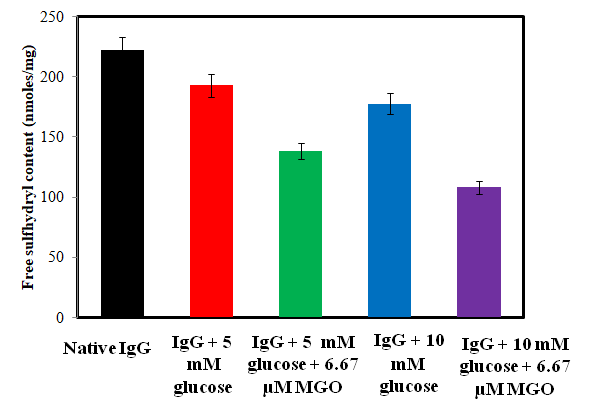
**

*

*

*

*

**Fig. C**

**
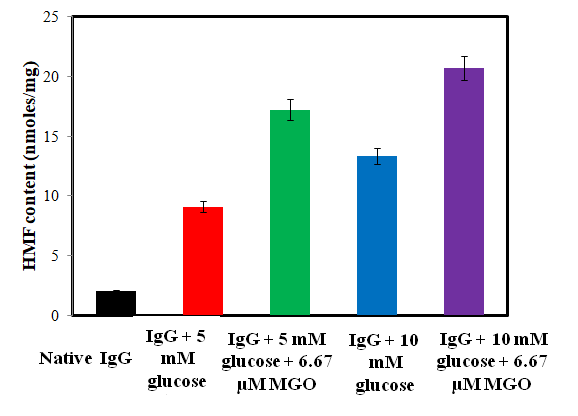
**

*

*

*

*

**Fig. D**

**
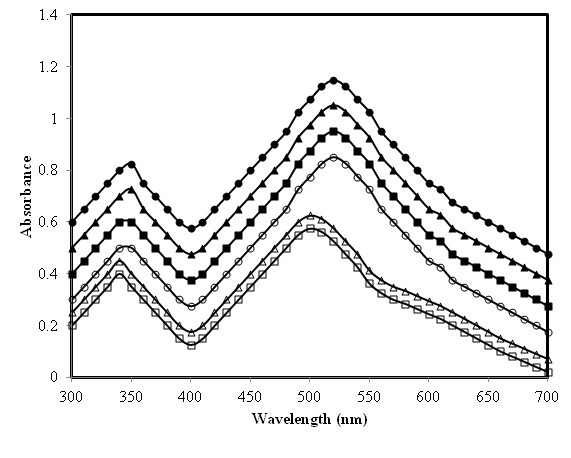
**

**Fig. E**

**
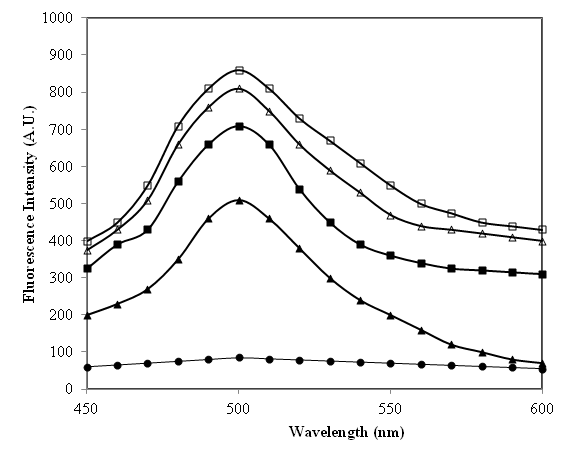
**

**Fig. F**

Supplement: S1 File — Fig A. Absorbance study. UV absorption spectra of native IgG (closed square) isolated from a healthy human serum on protein A-agarose affinity matrix. Inset: SDS-gel photograph of purified IgG on 7.5% polyacrylamide gel. Fig B. TNBS assay. Estimation of ε-amino groups in native IgG (black bar), IgG + 5 mM glucose (red), IgG + 5 mM glucose + 6.67 μM MGO (green), IgG + 10 mM glucose (blue), and IgG + 10 mM glucose + 6.67 μM MGO (purple). Each bar represents the mean ± S.D. of three independent assays in similar experimental conditions. * p < 0.05 was considered as statistically significant as compared to native IgG with each modified group. Fig C. DTNB assay. Free sulfhydryl in native IgG (black bar), IgG + 5 mM glucose (red), IgG + 5 mM glucose + 6.67 μM MGO (green), IgG + 10 mM glucose (blue) and IgG + 10 mM glucose + 6.67 μM MGO (purple). Each bar represents the mean ± S.D. of three independent assays in similar experimental conditions. * p < 0.05 was considered as statistically significant as compared to native IgG with each modified group. Fig D. HMF assay. Hydroxymethylfurfural content in native IgG (black), IgG + 5 mM glucose (red), IgG + 5 mM glucose + 6.67 μM MGO (green), IgG + 10 mM glucose (blue) and IgG + 10 mM glucose + 6.67 μM MGO (purple). Each bar represents the mean ± S.D. of three independent assays in similar experimental conditions. * p < 0.05 was considered as statistically significant as compared to native IgG with each modified group. Fig E. Congo Red binding assay. Absorption profile of Congo red (open square) bound to: native IgG (open triangle), IgG + 5 mM glucose (open circle), IgG + 10 mM glucose (filled square), IgG + 5 mM glucose + 6.67 μM MGO (filled triangle) and IgG + 10 mM glucose + 6.67 μM MGO (filled circle).Fig F. Thioflavin T binding assay. Emission profile of Thioflavin T bound to native IgG (filled circle), IgG + 5 mM glucose (filled triangle), IgG + 10 mM glucose (filled square), IgG + 5 mM glucose + 6.67 μM MGO (open triangle) [file pone.0191014.s001.docx]
